# RNA binding protein, tristetraprolin in a murine model of recurrent pregnancy loss

**DOI:** 10.18632/oncotarget.12539

**Published:** 2016-10-09

**Authors:** Kasra Khalaj, Rayana Leal Luna, Maria Eduarda Rocha de França, Wilma Helena de Oliveira, Christina Alves Peixoto, Chandrakant Tayade

**Affiliations:** ^1^ Department of Biomedical and Molecular Sciences, Queen's University, Kingston, ON, Canada; ^2^ Ultrastructure Laboratory, Aggeu Magalhães Research Center of the Oswaldo Cruz Foundation in Recife - FIOCRUZ, Recife, PE, Brazil

**Keywords:** pregnancy, inflammation, RNA, RPL, 3′ UTR, Pathology Section

## Abstract

Recurrent pregnancy loss is a major reproductive pathology affecting 1-5% of pregnant women worldwide. A distinct feature of this reproductive pathology is involvement of key inflammatory cytokines and transcription factors such as tumor necrosis factor alpha (TNF-α), interleukin 6 (IL-6) and nuclear factor kappa beta (NF-κB). Special classes of RNA-binding proteins regulate the transcripts of many of these important cytokines and regulatory factors via binding to the 3′ untranslated regions (UTRs) and/or poly(A) tail and destabilizing/stabilizing the transcript. The tristetraprolin (TTP/*ZFP36*) family have been found to be potent destabilizers of the aforementioned inflammatory and cellular response cytokines. The aim of this study was to evaluate whether tristetraprolin is expressed in the placenta and involved in modulating inflammation in mouse model of lipopolysaccharide (LPS)-induced fetal loss. In this study, Swiss-albino mice were injected with LPS at gestational day 15.5 and placental tissues were harvested 6 hours post-LPS injection. Histopathology and immunohistochemistry analyses clearly revealed cellular stress and death in LPS treated placentas compared to controls. TTP protein was downregulated, while targets TNF-α and IL-6 were upregulated in LPS group compared to controls. We observed increased TTP nuclear immunolocalization corresponding with higher NF-κB nuclear localization in trophoblasts from LPS treated placentas. Our results suggest that RNA-binding proteins such as TTP are expressed and perhaps involved in the modulation of inflammation-induced pregnancy pathologies.

## INTRODUCTION

Recurrent pregnancy loss (RPL) has been found to affect up to 5% of women of reproductive age worldwide [1, 2]. This multifactorial disorder has been classified for patients who experience pregnancy failure in the first trimester more than two or three times [1, 2]. Inflammation has been found to be closely linked with thrombotic events during fetal loss as well as fetal distress and indeed, both of the molecular pathways can directly influence the fetal health outcome [3, 4]. LPS-induced inflammation during pregnancy has also been shown to increase maternal inflammation as well as thrombosis status [5].

Successful embryo implantation requires a delicate balance of inflammatory changes in the endometrium [6-8]. Dysregulation of the early inflammatory processes have been found to contribute to pregnancy loss, due to its importance for early embryo growth and implantation [9]. During the first-trimester of human pregnancy, T helper (Th) type 1 inflammation, which includes inflammatory cytokines tumor necrosis factor alpha (TNF-α) and interferon gamma (IFN-γ) is predominant [7]. However, as pregnancy progresses and second-trimester begins, the dominant Th 1 type shifts to type 2 which includes interleukins (IL) 4, IL-10 immune responses which triggers maternal immune tolerance to the allogenic fetus [7]. In many cases of RPL, higher levels of Th1 cytokines have been found to persist, which is believed to contribute to allogeneic fetal rejection [10]. Due to this dysregulatory environment, both Th1 type cytokines TNF-α and IFN-γ have been found to play roles in spontaneous abortion/RPL [11, 12].

The tristetraprolin family of zinc-finger proteins are commonly known as RNA binding proteins which carry out mRNA destabilizing actions [13]. This gene family is composed of zinc-finger protein 36 (*ZFP36*/TTP), zinc-finger protein 36-like 1 (*ZFP36L1*/Tis11b), zinc-finger protein 36-like 2 (*ZFP36L2*/Tis11d), and the less studied rodent-specific *ZFP36L3*. Together, this gene family has been found to bind to transcripts of many prolific cytokines involved primarily in inflammation, cellular maintenance, proliferation and apoptosis pathways [14-16]. Primary transcripts that the tristetraprolin family has been found to regulate include: TNF-α [17], granulocyte-macrophage colony stimulating factor (GM-CSF) [18], IFN-γ [19], as well as nuclear factor kappa beta (NF-κB)[20], and chemokines including CXCL1 [21, 22]. This gene family can mediate degradation of its target transcripts via binding to AU rich element regions (AREs) that reside within the 3′ untranslated regions (UTR) [23], or via ARE-independent poly(A) tail mediated degradation or suppression [24]. It can shuttle rapidly between the cytoplasm and nucleus, and with the exception of ZFP36L3, is expressed in both cellular areas [23, 25]. This gene family has been previously shown to be required for successful embryo development [26]. Mice deficient in *ZFP36L1* do not undergo adequate chorioallantoic fusion and thus *ZFP36L1* is embryonic lethal [26]. Deficiencies of the rodent-specific, placenta and yolk sac-specific *ZFP36L3* have also shown that intrauterine deficiencies of these RNA binding proteins can result in major deleterious outcomes in the offspring [25]. In addition, TTP is now classified as a global post-transcriptional regulator of inflammation [27]. On the other hand, Human-antigen R (HuR/ELAVL1) is a potent gene stabilizer [28]. Working in a similar mechanism of action to the tristetraprolin family, HuR acts in an opposite manner to TTP, and instead can stabilize the same targets possessed by TTP. Previously, HuR has also been established to be crucial for embryonic development via its critical role in placental branching and morphogenesis [29]. Thus, in many non-reproductive pathologies, these groups of RNA binding proteins require a delicate homeostatic balance, and dysregulation of this imbalance may lead to further development of RNA binding protein associated pathologies [15, 30, 31].

Our group has previously shown therapeutic benefit of usage of phosphodiesterase-5 inhibitor alone or in combination with low molecular weight heparin as potentially having direct benefits in a similar murine model of spontaneous abortion/RPL [32]. We have also shown that the tristetraprolin family may be involved in spontaneous fetal loss in a porcine model of pregnancy loss [33]. In other animal models, inflammation has been related to an impairment in spiral artery remodelling [34]. Studies have investigated similar RNA binding proteins in other human-associated reproductive tract pathologies such as endometriosis [35, 36]. Therefore, our aims were to 1) characterize expression of tristetraprolin in murine placental trophoblast cells and to 2) investigate whether RNA binding proteins are involved in a mouse model of LPS-induced abortion at the placental site.

## RESULTS

### Histopathology analysis of placenta at gd 15.5 in murine model of RPL

**Figure 1:**
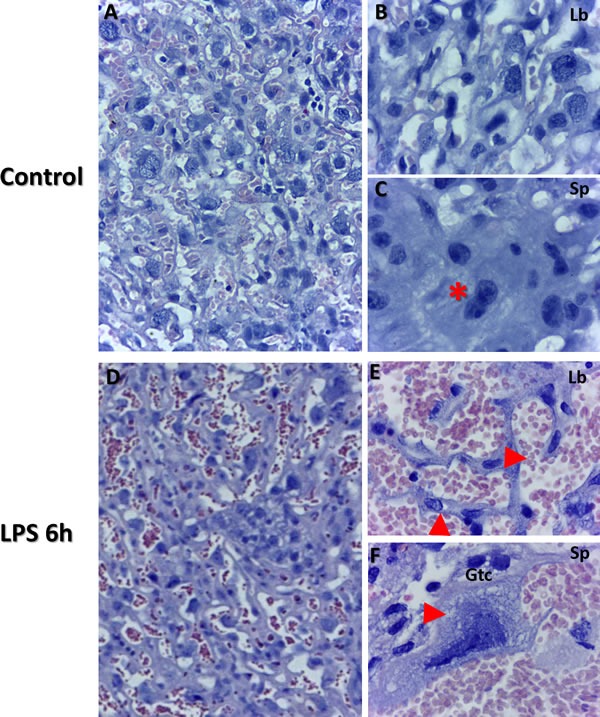
Placental histopathology using Haematoxylin and Eosin staining of control and LPS groups in mice at gd 15.5 Morphology was examined from both control (Panels **A.**-**C.**, *n* = 6), and LPS post 6 h (Panels **D.**-**F.**, *n* = 6) group placentas. Panel B illustrates labyrinth histology including giant trophoblast cells and maternal/fetal blood vessels. Panel C illustrates fusing binuclear giant trophoblast cells (red star). Disrupted blood vessels are evident in panel E (top red arrow). Additionally, disrupted trophoblasts are also evident in LPS group placentas (bottom red arrow in panel E). Panel F illustrates trophoblast cell (gtc) with evidence of vacuolization (red arrow) and nucleolar degradation (Panel A&D, 50 μm). Panels B-C, E-F (20 μm).

Haematoxylin and eosin staining revealed larger disruptions in tissue architecture in labyrinth layer of placenta as well as in giant trophoblast cells from the LPS compared to control groups. Placental villi appeared to be filled with blood, indicating vascular malformations in the LPS treated placenta (Figure 1; Panel E) compared to control, which is typical evidence of cell death and cell dispersion for abortion models in rodents (Figure 1; Panels A and D, Panels B and E). Histological architecture of the control placenta relevant to specific gestation day served as a control. Giant trophoblast cells imaged in the LPS treated group exhibited signs of nuclear damage and vacuolization (Figure 1; Panel F). These findings further confirm validity of LPS as a potential inducer of inflammatory response and associated impact on the placental vasculature [39, 40].

### Immunohistochemical assessment of cellular stress

**Figure 2:**
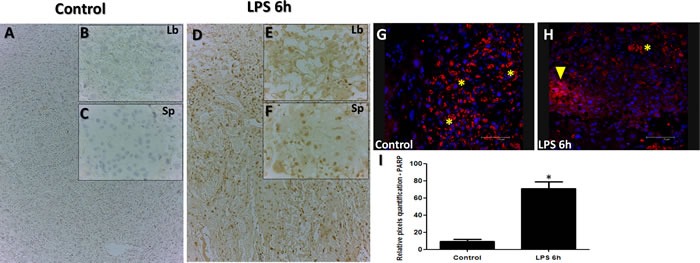
PARP and Caspase staining in placenta from LPS treated group compared to control PARP staining in Control (Panels **A.**-**C.**) and LPS **D.**-**F.** groups. PARP staining is significantly higher in placenta from LPS treated group compared to control (Panels A-F). Caspase-3 immunofluorescence analysis reveals staining in control (Panel **G.**) and LPS treated (Panel **H.**) and indicative of trophoblast foci in damaged placental region (H; yellow arrow that are reactive in trophoblasts treated with LPS compared to control). Asterisks in G&H indicate non-specific staining marked by red blood cells from the labyrinth region. Significantly high intensity of PARP staining was detected in LPS group (Intensity quantified using pixel quantification software (panel **I.**)). Panel I; Histogram results are presented as mean of relative pixel quantification ± SEM. Images (panels B&C, E&F, 20 μm (panels A&D, G&H, 100 μm). * *P* < 0.05.

In order to determine extent of apoptosis and cell death in response to LPS injection, we used immunohistochemistry for Poly (ADP-ribose) polymerase (PARP), a nuclear enzyme involved in DNA repair (a well-known substrate for caspase-3 cleavage during apoptosis). PARP staining analysis revealed higher staining intensities of PARP in both labyrinth (Figure 2; Panels E compared to B), and spongiotrophoblast (Figure 2; Panels F to C) placental regions, confirming cellular stress. Spongio-labyrinth placental regions from LPS group also exhibited significantly higher intensity of PARP immunostaining compared to controls (Figure 2; Panel I, *p* < 0.005). To further strengthen our observation of cellular stress and apoptosis, caspase-3 was also assessed using immunofluorescence. Higher Caspase-3 staining was observed in Trophoblast foci from specific damaged regions of placenta in LPS treated group compared to control (Figure 2; Panel H).

### NF-κB and TTP immunolocalization in placenta

**Figure 3:**
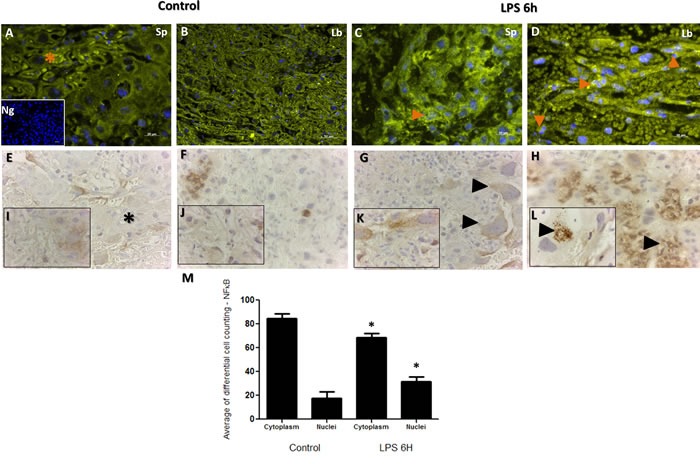
Immunohistochemical localization of NF-κB in placenta from LPS treated and control groups Panels **A.** and **C.** illustrate NF-κB staining in spongiotrophoblast, and panels **B.** and **D.** illustrate labyrinth region of the placenta. Similar to TTP, staining is noted in both cell cytoplasm (A; orange star) and nucleus (C&D; orange arrows), with higher cytoplasmic staining noted in control group compared to LPS group (orange star; panel A). Higher nuclear localization of NF-κB corresponded to TTP findings in figure 4; whereby significantly higher NF-κB staining was also noted in LPS group compared to control (evident in trophoblast cell in panel B; spongiotrophoblast region). Isotype negative control image is included in lower left of panel A. Panels **E.** and **G.** illustrate non-phosphorylated NF-κB staining in the cell cytoplasm, and phosphorylated NF-κB staining in the trophoblast nucleus (Panels **F.** and **H.**; panel **L.** black arrow at higher resolution). Similar to Panels A-B immunofluorescence findings, non-phosphorylated NF-κB are detected in similar quantities in trophoblast cytoplasm in LPS group (Panel G arrows) compared to control (Panel E star). Higher phosphorylated NF-κB nuclear localization is found in many trophoblast foci of LPS group compared to control (H&L compared to F&J). Histogram results (Panel **M.**) of panels A-D are presented as mean or average of differential cell counting ± SEM. * *P* < 0.05. Original magnification 20 μm (Panels A-D, **I.**-**L.**), 50 μm (Panels E-H)

NF-κB has long been a prototypical, well established pro-inflammatory signaling pathway, largely based on the activation of NF-κB by pro-inflammatory cytokines such as interleukin 1 (IL-1) and TNF-α. Furthermore, NF-κB signaling pathway is established to down-regulate the TTP expression during LPS-induction in macrophages [41]. In order to establish the extent of placental inflammation and cellular death in response to LPS injection and cross talk between NF-κB and TTP, we performed immunohistochemistry for NF-κB and TTP in the placenta samples obtained from LPS treated and control groups. Subcellular localization analysis for NF-кB exhibited no major differences in staining intensity in trophoblast cells as illustrated in Figure 3 Panels A-D. In trophoblast cells, NF-κB and TTP are normally expressed in both the cytoplasm and nucleus. Aberrant nuclear localization of NF-κB has been documented in human carcinoma pathologies [42]. Indeed, we observed this staining pattern, but noted significant nuclear localization of NF-κB observed in trophoblast cells residing in both spongiotrophoblast (Figure 3; Panel C orange arrow), and labyrinth (Figure 3; Panel D orange arrows) placental regions in LPS treated group compared to control. The localization characteristics of NF-κB was further stratified by immunohistochemical staining of phosphorylated and unphosphorylated forms of NF-кB. Indeed, higher unphosphorylated NF-кB staining was observed in cytoplasm from LPS treated trophoblast cells compared to control (Figure 3; Panel G compared to E, higher magnification Panel K compared to I). Additionally, higher phosphorylated NF-кB staining was observed in nucleus from LPS treated trophoblast cells compared to control (Figure 3; Panel H compared to F, higher magnification Panel L compared to J). The immunofluorescence localization findings were further quantified via differential cell counting of trophoblast cells (Figure 3; Panel M), and resulted in a decreased cytoplasmic NF-kB and increased relative nuclear staining ratio in trophoblasts in the LPS compared to control groups (*p* < 0.05).

**Figure 4:**
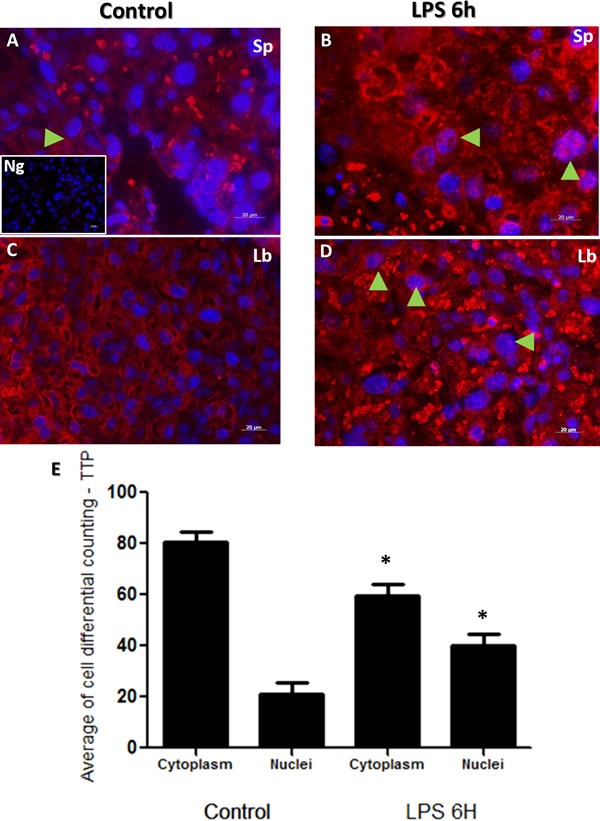
Immunohistochemical localization of TTP in placenta from LPS treated and control groups TTP staining in spongiotrophoblast (panels **A.** and **B.**), and labyrinth (panel **C.** and **D.**) region of the control (panel A and C) group and LPS challenged (panel B and D) placentas. Cytoplasmic and nuclear TTP staining was observed, especially in labyrinth area in control placenta. However, higher nuclear localization of TTP is noted in trophoblast and giant trophoblast cells residing in the spongiotrophoblast region of LPS group placentas (panel B, arrow indicate trophoblast cells). TTP is also expressed in labyrinth region of placentas in LPS treated mice (panel D, arrows emphasize nuclear staining). Isotype control image is illustrated in lower left of panel A. Panel E histogram presented as mean or average of differential cell counting ± SEM. * *P* < 0.05. Original magnification 20 μm.

Immunofluorescent images showed similar staining intensities for TTP protein in murine placental cellular microarchitecture as illustrated in Figure 4 Panels A-D. Similar to NF-κB protein immunolocalization findings in Figure 3, TTP immunolocalization was observed to be higher in trophoblast nuclei from both spongiotrophoblast and labyrinth placental regions of LPS treated groups (Figure 4 Panels B and D; total counting in both spongeotrophoblast and labyrinth regions). In a similar manner to NF-κB, TTP differential cell counting revealed significantly higher TTP nuclear staining in trophoblast cells from LPS compared to control groups (Figure 4; Panel E, *p* < 0.05).

### TTP localization using immunogold electron microscopy in placenta

**Figure 5:**
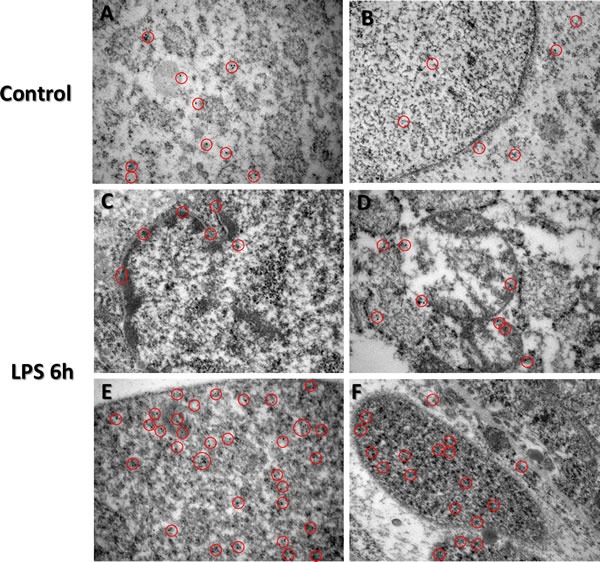
Immunogold-EM analysis of TTP expression in LPS treated and control groups Immunocytochemistry (ICC) images of TTP expression via immunocytochemistry in control (Panels **A.** and **B.**) and LPS (Panels **C.**-**F.**) group placentas reveal unique nuclear staining patterns. Cytosolic immunogold labelled TTP is evident in varying trophoblast cells from the spongiotrophoblast and labyrinth regions of both LPS and control group placentas (Panels A-D). Higher immunogold labelled TTP is bound to nuclear content within the trophoblast nuclei in the LPS group compared to control (panels C&E). Red circles indicate immunogold staining specific for TTP. Original magnification 500 nm.

Immunogold labelled TTP particles were observed to be bound within the cytosol compared to cellular nucleus in control group placental trophoblast cells (Figure 5; panel A cytosolic staining compared to panel B nuclear staining). However, higher than normal quantities of immunogold labelled TTP were identified in nuclei of trophoblast cells in LPS group (Figure 5; panel E and F compared to B), compared to the control group. Higher immunogold labelled TTP was also observed to be bound to the nuclear content within the trophoblast nuclei in the LPS group compared to control (Figure 5; panels C&E). Cytosolic immunogold labelled TTP binding was also observed (Figure 5; panel C), with higher concentration of immunogold labelled TTP observed at the nuclear membrane (Figure 5; outlined in panel C). Additionally, immunogold labelled TTP was observed in degrading mitochondria (Figure 5; panel D).

### Placental ultrastructure analysis

**Figure 6:**
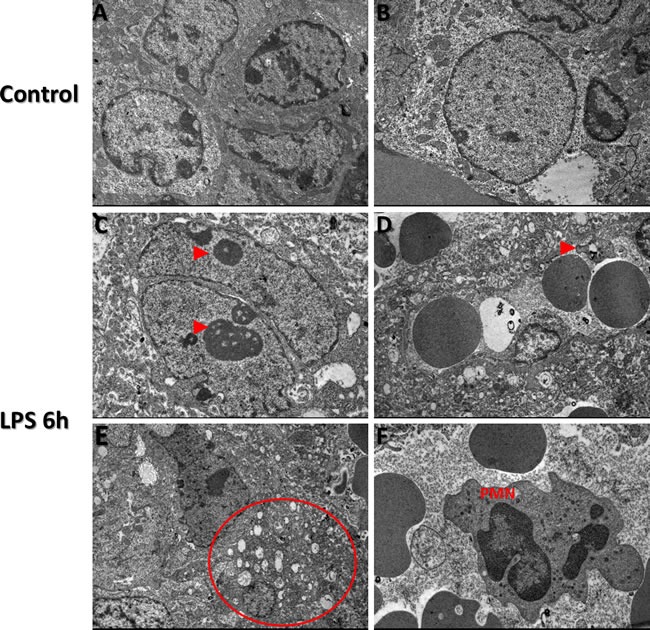
Transmission Electron Microscopy images of TTP expression via in LPS treated and control groups Transmission electron microscopy findings in control (A and B) and LPS (Panels C-F) treated placental trophoblast cells Ultrastructural abnormalities and defects existed in placental tissue in LPS group compared to control and also exhibited signs of dysmorphic placental tissue microarchitecture (Red arrows, panels **B.** and **E.**). Enlarged nucleoli are present in residing trophoblast nuclei from LPS treated placentas (Red arrows, panel **C.**). Figure 5D, red arrow indicates presence of a platelet in LPS group compared to control. Vacuolization of cytoplasm is confirmed via observed cytoplasmic vacuolization (Circled region, panel E, which also corresponds to our histopathology findings in LPS group compared to control. Additional findings included Polymorphonuclear bodies (PMN) observed in low quantities in LPS group placentas compared to control (Panel **F.**). TEM images were obtained at 2 μm.

Immune cell screening revealed absence of immune cells in all control group placenta TEM grids analyzed. Enlarged nucleoli were present in residing trophoblast nuclei from LPS placentas, characterizing nuclear condensation (Figure 6; panel C, arrows). As well, these enlarged nucleoli were more often observed in trophoblast cells from LPS treated group when compared to control group (Figure 6; panels C&B). Disorganization of ultrastructure in placental tissue was evident in LPS group compared to control (Figure 6; Panel D). In LPS group, we also observed presence of platelets (Figure 6; panel D, arrow). Vacuolization of cytoplasm was a striking feature in LPS treated placenta compared to control, corresponding to the histopathology findings (Figure 6; Panel E). Polymorphonuclear bodies (PMN) were also observed in LPS group placentas compared to control (Figure 6; panel F) further confirming LPS-induced inflammation. Additionally, trophoblast nuclei appear dysmorphic in nature in LPS group placentas compared to control.

### Characterization of the TTP and its associated target proteins (IL-6 and TNF-a) in placenta

**Figure 7:**
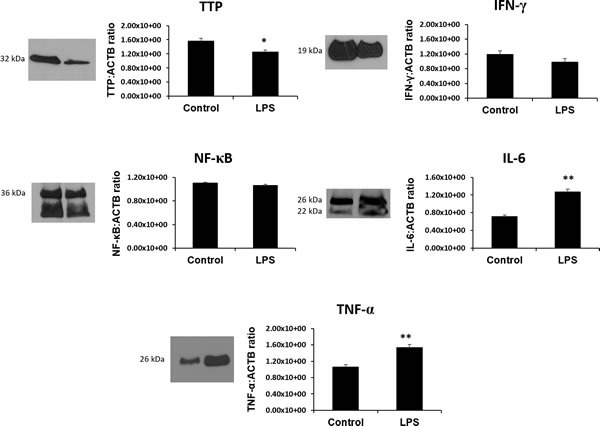
The RNA binding protein TTP is significantly downregulated and its inflammatory targets IL-6 and TNF-α are significantly upregulated in LPS 6h treated placentas Protein expression of other TTP-associated genes and targets IFN-γ, NF-κB while slightly lower, remain unchanged in LPS compared to control groups. All data is expressed as a ratio to corresponding ACTB expression. Expression data illustrated as mean ± SEM. * *P* < 0.05, and ** *P* < 0.005.

Using western blot, a single band at 32 kDa was quantified and determined to be TTP based on the manufacturer's suggested band size that is characteristic of TTP protein (Figure 7) [43]. Significantly lower TTP protein was observed in the LPS group compared to control (*p* < 0.05). In contrast, IL-6 (confirmed double bands at 26 kDa and 22 kDa) and TNF-α (26 kDa) protein was significantly higher in LPS group compared to control (*p* < 0.05) [44,45]. IFN-γ band at 19 kDa (abcam confirmed molecular weight) and NF-кβ (36 kDa abcam confirmed molecular weight and unidentified secondary band) proteins, while slightly lower, was not found to be statistically significant.

### mRNA profile characterization of RNABPs in placenta and HTR8 SVneo

**Figure 8:**
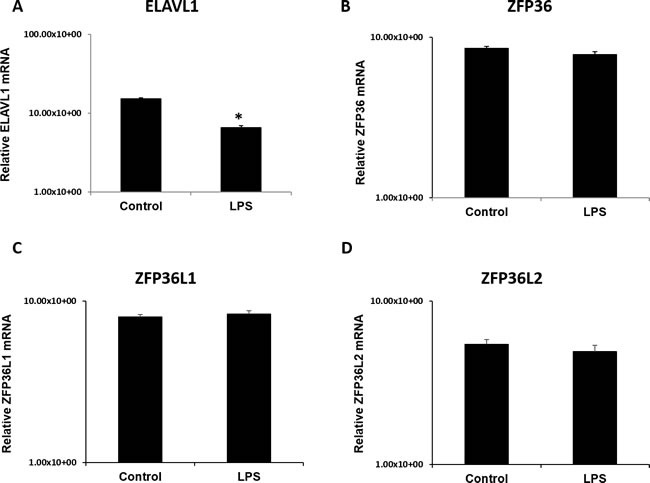
ELAV like RNA binding protein 1 (ELAVL1) mRNA expression is significantly lower in LPS 6h treated placentas compared to control mRNA transcripts of destabilizing RNA-binding proteins *ZFP36*, *Z*FP36L1, ZFP36L2 (panels B-D) remain unchanged in LPS treated placentas compared to control. All data was normalized and expressed relative to GAPDH housekeeping gene. Expression data illustrated as mean +/− SEM. * *P* < 0.05.

To understand the interplay between cytokine destabilizers (TTP/*ZFP36, ZFP36L1, ZFP36L2*) and recently emerged stabilizers (*ELAVL1*) for the same cytokine targets (IL-6, TNF-α), we investigated expression of ELAV1 at the mRNA level. Transcripts for *ELAVL1, ZFP36, ZFP36L1*, and *ZFP36L2* were found to be expressed in the placental samples from both LPS and control groups (Figure 8). Transcript levels did not vary between biological replicates for all genes of interest Figure 8. Significantly lower *ELAVL1* was detected in LPS group compared to control (Figure 8; panel A, *p* < 0.05). Transcripts for *ZFP36*, *ZFP36L1*, and *ZFP36L2* did not statistically differ between control and LPS groups (Figure 8, panels B-D). Additionally, transcripts for *ELAVL1, ZFP36*, and *ZFP36L1* were measured in our *in vitro* model LPS stimulated HTR8 cells and compared with control at 6 h, 12 h and 24 h time points (Supplemental Figure 2). Transcript levels of *ZFP36, ZFP36L1 and ELAVL1* were significantly upregulated at 6 h at 100 ng treatments (*p* < 0.05; Supplemental Figure 2; Panels A-C). This was also observed at 12 h at both 10 ng and 100 ng treatments for *ZFP36* and *ZFP36L1* (*p* < 0.05; Supplemental Figure 2; Panels A and B). Lastly, transcript levels for all three genes were significantly upregulated at 24 h at both doses, with the exception of *ZFP36L1*, which differed only at 10 ng (*p* < 0.05; Supplemental Figure 2; Panels A and B).

### *in vitro* analysis of TTP in cultured first trimester trophoblast cells, HTR8 SVneo

In order to validate our immunolocalization experiments in our *in vivo* LPS model, the first trimester trophoblast cell line, HTR8 SVneo was utilized. TTP immunolocalization was detected with laser scanning confocal microscopy in both nucleus and cytoplasm of LPS treated and control groups (Supplemental Figure 3, panels A-I). Higher nuclear as well as cytoplasmic TTP were detected in 10ng (B,E,H) and 100ng (C,F,I) treated groups compared to their respective time controls (A,D,G). Higher TTP fluorescence is detected in 100ng LPS treated group when treated for longer 12h and 24h time points (Supplemental Figure 3, panels F compared to D and I compared to G), which complements our *in vivo* murine model data.

## DISCUSSION

Throughout gestation, a constantly changing inflammatory microenvironment composed of Th1 and Th2 cytokines are required to allow for embryo attachment and to mediate pregnancy [46]. However, our understanding of exactly the balance of levels of Th1 and Th2 type inflammatory cytokines required to modulate a successful pregnancy is not yet fully understood. While acknowledging other contributing factors for RPL (immune cells, BMI, allogeneity), we sought to characterize RNA binding proteins in relation to imbalanced levels of classical inflammatory cytokines in placentas from aborting fetuses and to compare and contrast to homeostatic cases which ultimately lead to successful pregnancy outcomes. Based on our previous work with associations of RNA binding proteins with spontaneous fetal loss in pigs, we hypothesized that these RNA binding proteins will be differentially expressed in a LPS induced abortion model in mice. We observed a downregulation of TTP in LPS treated placentas, which suggests to us that this protein is implicated in aberrant maternal-inflammation cases of recurrent pregnancy loss. We also accept the possibility that the observed TTP downregulation may also be due to the fact that we collected our placentas at 6 hours post-LPS injections. As previously documented in a rat model, TNF levels peak at approximately 2 hours of LPS-injection and decline after 6 hours in rodents [39]. This decline corresponds with increased coagulation and thrombotic events [39]. With TNF being a major target of TTP, and TTP interactions via TNF and nuclear factor 3′ binding sites being classified among the highest in bioinformatics screenings [27], it is logical that TTP levels will decrease as they along with other potential destabilizing RNA binding proteins may bring down levels of TNF in a feedback manner. Further, TTP has been shown to be a global post-transcriptional regulator of inflammation [27] and our results suggest that it may be participating in modulating inflammation in the placenta.

In our murine model of pregnancy loss, we demonstrated increased cellular apoptosis and stress that occur in spongiotrophoblast and labyrinth regions in the LPS treated placentas. In addition, our histopathology analysis yielded similar findings to other models of pregnancy-associated loss, whereby elevated TNF levels in the placentas treated for 6 hours with LPS were observed in rats [39]. Our initial pilot studies have also unravelled higher elevated TNF levels in placentas treated with LPS for 2 hours (unpublished data), which further corresponds to previously documented studies of pregnancy loss [39, 47]. Together, these findings provide us with evidence that our murine model is indeed, adequately mimicking inflammation-associated pregnancy loss via aberrant maternal inflammation.

Recently, rodent-specific ZFP36L3 has been shown to be expressed abundantly in the murine placenta [25]. Due to their functional differences, the other members of the TTP family do not possess such unique expression patterns. In normal physiological conditions, NF-κB and TTP have been shown to be expressed in both cytoplasm and nucleus of a wide variety of cell types, with macrophages being the most widely studied for the TTP family [23]. While aberrant NF-κB has been linked with a variety of cancers in humans, TTP immunolocalization has never been documented in the placenta. In 293 or HeLa cells, TTP and TIS11d have been found to be expressed primarily in the cellular cytoplasm, but are also expressed in low levels in the nucleus [23]. Indeed, one of our most interesting findings was the corresponding higher TTP nuclear staining and NF-κB in trophoblast cells residing in both the spongiotrophoblast and labyrinth regions in LPS challenged placentas. We also observed higher TTP nuclear staining in the invasive HTR8 SVneo trophoblast cell line when stimulated with varying doses of LPS, which complements our findings in our *in vivo* mouse model. Based on our immunofluorescence, *in vitro* based and electron microscopy experiments, TTP is binding in a dysregulated fashion in these LPS challenged placentas. To further support our findings, it has been previously published that TTP and NF-κB share binding domains and that cellular stimulation by LPS can trigger NF-κB signaling mechanisms to activate in order to regulate the transcription of TTP mRNA [41]. In a recent study, TTP interactions with NF-κB domains were classified as being one of the highest global associations in modulating feedback-inhibition of inflammation [27]. TTP family members have been shown to be able to nucleocytoplasmic shuttle via CRM1 [23]. Additionally, it has been established that TTP degradative roles are not only via cytosolic mRNA binding, but can also be via inhibition of poly(A) tail synthesis via TTP interaction with poly(A) binding protein nuclear 1 in the cell nucleus [24]. We believe that these findings when taken together can be correlated to the dysregulation in TTP as well as inflammatory cytokine protein observed from our western blotting results. When taken together with the established literature, we believe that one of these mechanisms may necessitate a higher role for poly(A) binding protein mediated mRNA degradative functions compared to traditional 3′ UTR mediated degradative functions previously established as TTP's primary role.

Reproductive tract pathologies, including RPL include subsets of patients that undergo pregnancy loss due to dysregulation of Th1 maternal-inflammation. Setting aside other factors identified above as causes of RPL, and using a targeted focus on inflammation-induced cases of RPL in particular, we believe that this dysregulation of RNA binding proteins and inflammatory targets at the protein level at the site of the placenta may either be a cause or an effect of this subset of RPL pathologies. There exists the possibility that a dysregulation of RNA binding proteins that can destabilize vs stabilize these inflammatory cytokines may be causing the inflammatory cascade previously published. On the other hand, this dysregulation may also be an effect observed. Nevertheless, further studies will need to be conducted to examine this causality, including immunoprecipitation and/or use of luciferase target reporter assays.

Recent literature suggest a direct functional link between RNA-binding proteins and microRNAs, or small classes of RNAs that have similar regulatory roles as RNABPs [31, 48, 49]. In light of these findings, further studies will need to be conducted to not only investigate RNA stability via RNABPs, miRNAs, and other classes of RNA regulators, but with other classes of regulators as well. Our mRNA results of the TTP family did not yield any differences in the murine model, compared to the dysregulation in TTP protein, suggesting a possibility for post-transcriptional modification of *ZFP36* may be at play. We observed ratio changes between mRNA stabilizers (*ELAVL1*) and mRNA destabilizers (TTP family of transcripts; *ZFP36*), whereby there is lower ratio of *ELAVL1* transcripts compared to *ZFP36* and other TTP family members. This may be one of the unique ways that inflammation is modulated in the placenta. Since HuR (*ELAVL1*) can also bind to *TNF-α*, it's transcriptional repression, it is logical to assume why TNF-α levels drop after a peak at 2 h, which allow for thrombotic events to initiate. Furthermore, in our *in vitro* based experiments with HTR8 SVneo trophoblast cells, we saw drastic differences in mRNA for all RNA binding proteins in the cells treated with LPS. We believe we may not be seeing the same changes in our mouse model due to the complexity that *in vivo* models bring. In the invasive HTR8 SVneo trophoblast cells, there are no interactions with endothelial cells, immune cells and no maternal-fetal interface is present which may explain why we see clearer changes at the mRNA level. Additionally, we believe that the aforementioned microRNA effects on RNA binding proteins may be a potential reason to explain for this discrepancy between mRNA and protein expression levels. While not the main focus or outcome of this study, future studies in our laboratory will seek out to explore the unique potential interplay between microRNAs and RNA binding proteins to help us to further elucidate this complex mechanism of regulation.

To the best of our knowledge, this work is the first to categorize the expression patterns of tristetraprolin in the context of inflammation in the placenta. These results lay down the groundwork for future studies investigating the role of RNA binding proteins in inflammation-mediated reproductive tract pathologies. We acknowledge that future studies will be necessary to determine the true causality of these findings, and that further characterization will need to be performed with regards to expression patterns of immune cells that have also been found to be playing a role in RPL. Further studies will need to be conducted in order to determine levels of specific pathways; 3′ UTR or non-ARE mediated Poly(A) suppression that can occur in the LPS treated placentas. Our group aims to further characterize these RNA binding proteins in the hopes of improving our understanding of them, and their contributions to reproductive tract pathologies.

## MATERIALS AND METHODS

### Mouse model of RPL

In order to mimic dysregulated maternal-inflammation cases of RPL, 18 pregnant Swiss albino mice (aged 60 days) were bred in-house (FIOCRUZ-PE, Recife, Brazil) and divided into two groups (Supplemental Figure 1). All animal procedures were performed in accordance with protocols approved by the Institutional Animal Care and Use Committee (IACUC) and Animal Care Committee of Aggeu Magalhaes Research Center (FIOCRUZ) protocol #P-408-68. Copulation was confirmed the morning after mating by the presence of a vaginal plug. Determination of a vaginal plug was considered day 0.5 of pregnancy. Mice were separated into two groups: control group (*n* = 9), and LPS treated group (*n* = 9). Mice in control group received 0.5 mL of saline intraperitoneally, while mice in LPS group received 100 μg/Kg of LPS intraperitoneally (Escherichia coli serotype, Sigma-Aldrich 0111-B4) on day 15.5 of pregnancy and euthanized 6 h post-injection. All implantation sites were grossly examined during time of collection for each group after 6 h of LPS injection and all appeared to be similar in size and colour. Tissue collection was performed on the pregnant females following euthanasia by anaesthesia overdose with ketamine (100 mg/kg) and xylazine (10 mg/kg) for all aforementioned groups of mice. Placenta samples were collected and were snap-frozen in liquid nitrogen and stored at −80°C.

### Histopathology analysis

The effect of LPS treatment on gd 15.5 placentas was assessed via histopathological analysis. Briefly, placental tissues were fixed in 4% buffered paraformaldehyde (PFA) for 24 h and dehydrated in an ethanol series, followed by xylene series and paraffin embedded. Microtome (Reichthert S, Leica, Rio de Janeiro, Brazil) sections (6 μm) were mounted onto glass slides and stained with hematoxylin-eosin and assessed at 200 X and 400 X magnifications using an inverted photomicroscope (Observer Z1, Zeiss GmbH). The presence of cellular and tissue damage indicators such as: vacuolization, nuclear compression, edema, cellular death, congestion, haemorrhagic foci were observed in detail and compared between control and LPS 6 h groups.

### Immunohistochemical assessment of cellular stress

Lipopolysaccharide and control placenta tissue samples from 6 animals per group were embedded in paraffin, cut (8 μm) and mounted on slides. Antigen retrieval was performed by boiling in citric acid buffer at pH 6.0 for 30 s. Sections were blocked in 0.1% bovine serum albumin (BSA) for 1 hour at room temperature. Anti- Nuclear factor-κB (NFkB) (ab31481, Abcam Plc, Cambridge, UK), NF-κB p65-p (phosphor-ab97726 Abcam Plc, Cambridge, UK) and Poly(ADP-ribose) polymerase (PARP) primary antibody (ab6079, Abcam Plc, Cambridge, UK) were added to the sections at a concentration of 1.2 μg/mL, 1.5 μg/mL and 1 μg/mL, respectively. The PARP antibody employed for this experiment is reactive with both 113 kDa PARP as well as 29 kDa cleaved fragments. A biotin-conjugated secondary antibody using an HRP-kit (K0690 DakoCytomation, USA) was added to all sections and incubated for 1 hour at room temperature. DAB was used as a chromogen for the reaction and reactions conducted for 1 min for each slide. Counterstaining were performed using the same slides with haematoxylin as per standard histology protocols. Images for all slides were taken using ZEISS imaging software (Zeiss, São Paulo, Brazil). Pixel analyses were performed as per our groups previously published protocol using GIMP 2.6.11 image analysis software [32]. Briefly, five different areas from five different slides for both experimental groups for PARP anti-body were chosen for pixel analysis. All images had a total of 2014×1536 pixels each and were all from the same magnification (400 X) and taken with objective numerical aperture of 0.65. Colour related to the positive stain was selected to perform the differential pixels as per previously published protocol.

### Immunofluorescence analysis in murine placentas treated with LPS

LPS (*n* = 6) and control (*n* = 6) paraffin-embedded placental tissue samples were used from the immunohistochemical section for immunofluorescence of TTP, NF-κB and Caspase-3. Briefly, antigen retrieval was performed on all slides by boiling in citric acid buffer at pH 6.0 for 30 s. Sections were blocked in 0.1% bovine serum albumin (BSA) for 1 h at room temperature. Primary Anti-ZFP36/TTP (sc-12563, Santa Cruz Biotechnologies, Inc., Dallas, TX), Anti-NF-κB (ab31481, Abcam Plc, Cambridge, UK), and Anti-Caspase-3 (ab4051, Abcam Plc, Cambridge, UK) antibodies were added at concentrations of 1 μg/mL, 5 μg/mL, and 0.5 μg/mL respectively, and incubated overnight at 4°C. Normal rabbit serum was added at a concentration of 0.5 μg/mL which served as an isotype negative control (#08-6199, Thermo Fisher Scientific, Canada). Cy3 secondary anti-rabbit antibody (F6257, Sigma-Aldrich, São Paulo, Brazil) was added to all sections and incubated for 1 h at room temperature. All sections were also stained with DAPI (Sigma-Aldrich, São Paulo, Brazil). Sections were visualized using a xenon light microscope equipped with Cy3 and DAPI filters (Zeiss, São Paulo, Brazil). For TTP and NF-kB differential cell counting analyses, ImageJ image analysis software was used. Briefly, 100 cells were manually counted from five areas from five different slides in each experimental group. All images had a total of 2014×1536 pixels each and were all from the same magnification (500 X) and were taken with objective numerical aperture of 0.7. Red blood cells were excluded from the analysis. Cells which presented primarily either nuclear or cytoplasmic staining patterns were considered as positive.

### Determination of TTP cellular localization in placenta using immunogold electron microscopy

Small fragments of placenta samples were fixed with a solution of 0.5% glutaraldehyde and 4% paraformaldehyde (0.1 M phosphate buffer), dehydrated using increasing series of acetone and embedded in LR White resin. Polymerization using LR white resin (Sigma-Aldrich, São Paulo, Brazil) was performed in a sealed UV chamber for 48 h. Semi-thin (800 nm) sections were taken to select the placental regions (spongiotrophoblast and labyrinth) to be analyzed. Placental regions of both LPS and control groups were cut ultra-thin (80 nm) with a diamond knife and placed on nickel grids. Sections were incubated for 30 min at room temperature in 0.02 M PBS, pH 7.2, containing 1% BSA and 0.01% Tween 20 (PBS-BT). The sections were then incubated for 1.5 h with primary antibodies against TTP (LS-B1572, LifeSpan Biosciences Inc., Seattle, WA) at dilution of 25 μg/mL, in PBS-BT. Sections were washed in PBS-BT and incubated with a secondary antibody, 10 nm colloidal gold-labelled goat anti-rabbit IgG at 25 μg/mL (G7402, Sigma-Aldrich, São Paulo, Brazil). For antibody control, sections were incubated only with the secondary gold-labelled marker. Sections were then counterstained with 5% uranyl acetate and lead citrate. Quantitative analysis was performed at average magnifications of 56,000 X or 42,000 X. Images were taken from 10 areas for each grid in both groups, and randomly chosen to compare the numbers of gold-labelled particles in control and LPS 6 h groups. Positive staining was considered from the presence of gold spheres either in the nuclei or in the cytoplasm. The location as well as the quantity of the immunogold staining were confirmed with a blinded analysis from two trained researchers from our group.

### Placental ultrastructure analysis (Transmission Electron Microscopy)

Placental fragments from each group were fixed in Karnovsky's solution, and postfixed in 1% osmium tetroxide, and processed using previously published methodology from our group [37]. All fragments were dehydrated with acetone series washes and embedded with SPIN-PON resin (Embed 812-Electron Microscopy Science, Washington, PA., USA). Six resin blocks from 6 different placentas from each group were cut semi-thin (0.5 μm), positioned on slides and were stained with toluidine blue for morphometric analysis. Peripheral spongiotrophoblast and central labyrinth areas were imaged and pre-selected with an Observer Z1 Zeiss light microscope (Zeiss GmbH, São Paulo, Brazil) at 400 X, and chosen for ultrathin sectioning. Ultrathin sections (70 nm) for each group and each region were placed on 300-mesh nickel grids, counter-stained with 5% uranyl acetate and lead citrate, and examined using a FEI transmission electron microscope (Tecnai Spirit Biotwin, FEI, Oregon, USA).

### Determination of TTP and target proteins (TNF, IFN and Il-6) expression using Western Blot

In order to examine protein levels of the TTP and its inflammation-related targets: TNF-α, NF-κB, IFN-γ and IL-6, western blotting was conducted. Briefly, total protein was extracted from LPS treated and control placentas (*n* = 6). Approximately 30 mg of tissue was excised from whole tissue and was placed in 1.5 mL microcentrifuge tube containing 0.3 g/mL of protease inhibitor cocktail (Sigma-Aldrich, São Paulo), in 200 μL of Phosphate buffered saline (PBS). The tissue was homogenized using a rotor-stator homogenizer on ice. The samples were then centrifuged at 4°C and supernatant was collected. Protein concentrations were determined using a bicinchoninic acid (BCA) assay (Sigma-Aldrich, São Paulo) as per kit instructions. The samples were normalized to a protein concentration of 10 μg/μL using PBS and subsequently stored at −80°C. Samples were denatured at 100°C for 5 minutes in a thermal cycler (Applied Biosciences, São Paulo). Samples with 5 ul of gel loading dye added were pipetted to appropriate wells of 12% gels (12 wells/20μL) and separated at 120 V for approximately 1.2 h. The transfer step was run at 100 V for 2 h. Upon completion of transfer, membranes were removed and rinsed in TBS-T and TBS, then blocked in 5% skim milk TBS-T solution overnight. 1.5 μg/mL of Rabbit anti-ZFP36 (LS-B1572, LifeSpan Biosciences Inc., Seattle, WA), 1.0 μg/mL Rat anti-IFN-γ (Abcam Ab24979), 2.5 μg/mL of Rabbit anti-NF-κB (Abcam, Ab31481), 1.1 μg/mL of Rabbit anti-IL-6 (Abcam, Ab6672) and 1.0 μg/mL of Rabbit anti-TNF-α (Abcam, Ab34674) antibodies were added as primary antibodies. Membranes were rinsed 2 X at 10 mins with TBS-T solution and 3 X at 5 mins with TBS solution. HRP conjugated goat anti-rabbit IgG 1:3000 (R&D, HAF008), and goat anti-mouse IgG 1:8000 (Sigma-Aldrich, A0168) secondary antibodies were added in 5% skim milk TBS-T solution to each of the membranes and incubated on a rocker at room temperature (RT) for 1.5 h. Enhanced chemiluminescence detection was completed with Pierce ECL Western blotting chemiluminescent substrate solution (Thermo Fisher Scientific, São Paulo) and imaged on a C-digit digital ECL blotting scanner (LI-COR, UniScience, São Paulo, Brazil). Membranes were stripped and re-probed for ACTB using 1.8 μg/mL of anti-ACTB mouse monoclonal antibody (Sigma-Aldrich, A2228), and incubated on an electric plate-rocker in a 4°C refrigerator for 12 h. Images were further analyzed using ImageJ software (NIH, Bethesda, MD) to obtain densitometry values.

### mRNA profiles of RNABPs (TTP family and ELAVL1) in murine placentas and HTR8 SVneo trophoblast cells

The mRNA of TTP family as well as the mRNA stabilizer, *ELAVL1* were assessed using quantitative real-time PCR (qPCR). Total RNA from LPS-treated and saline-treated placenta samples as well as cell lysates from HTR8 SVneo cultures was reverse transcribed using Superscript II reverse transcription kit (Life Technologies, São Paulo, Brazil) as per the manufacturer's protocol. Primers were designed using Primer3 software (http://frodo.wi.mit.edu/primer3/) from murine and human sequences available on NCBI's Nucleotide. Primer sequences are listed in Table 1. Real-time PCR was performed using plate-based ABI-7500 PCR System (Applied Biosystems Inc., São Paulo, Brazil). Experimental set-up was according to the MIQE guidelines [38]. Relative quantification was performed using *GAPDH* as a control gene. Expression of *GAPDH* did not differ across groups by one-way ANOVA. All samples were run in triplicates. The run protocol for all genes of interest used was the following: Denaturation: 95°C, 15 min; Amplification: 45 cycles: 95°C for 15 s, 55°C for 30 s, 70°C for 30 s; Melting Curve: 70-95°C, at a rate of 0.1°C per second. Data was analyzed using the ΔΔCt method.

**Table 1:** mRNAs Assessed by Real-Time PCR.

Gene Name	Primer	Product Size (bp)	GenBank Accession Number
*mZFP36*	for: 5′-CTCCTGCCGAAGGTCTACTA-3′	155	NM_011756
	rev: 5′-TGCCTCAAAGACAGGTGAGTC-3′		
*mZFP36L1*	for: 5′-CAAGGGTAACAAGATGCTCAACTAC-3′	222	NM_007564
	rev: 5′-GAGAAAGAGCGGTCTCGAAAG-3′		
*mZFP36L2*	for: 5′-GCTGCCACCTCCCTAAACTA-3′	190	NM_001001806
	rev: 5′-GCAATGAGCCCGTTATCA-3′		
*mELAVL1*	for: 5′-GGCTGGTGCATCTTCATCTAC-3′	176	NM_010485
	rev: 5′-GCCATTGCAGCTTCTTCATAGT-3′		
*hZFP36*	for: 5′-CATGGATCTGACTGCCATCTA-3′	279	NM_003407.3
	rev: 5′-GAAGTGGGTGAGGGTGACAG-3′		
*hZFP36L1*	for: 5′-TCTGCCACCATCTTCGACTT-3′	109	BT019468.1
	rev: 5′-TGCCCACTGCCTTTCTGT-3′		
*hELAVL1*	for: 5′-GTTCAGCAGCATTGGTGAAGT-3′	253	BT009793.1
	rev: 5′-TTCTACGTCCTTCTGGGTCAT-3′		
*mGAPDH*	for: 5′-AGGTCGGTGTGAACGGATTTG-3′	210	GU214026
	rev: 5′-TGTAGACCATGTAGTTGAGGTCA-3′		
*hGAPDH*	for: 5′-GAGTCAACGGATTTGGTCGT-3′	238	M33197.1
	rev: 5′-TTGATTTTGGAGGGATCTCG-3′		

### *in vitro* analysis of TTP in cultured first trimester trophoblast cells, HTR8 SVneo using confocal microscopy and qPCR approaches

TTP immunolocalization was assessed in an *in vitro* LPS model using the immortalized Human first trimester trophoblast cell line, HTR8 SVneo (gift from Dr. Charles Graham, Queen's University, Kingston, ON, Canada). Briefly, HTR8 cells were grown in T75 tissue culture flasks (Sarstedt, Germany) until 60% confluence in RPMI-1640 medium (Gibco, Canada) containing 5% fetal bovine serum (Thermo Fisher Scientific, Canada). LPS was added to cells at 10ng or 100ng doses and PBS was added to cells as a vehicle control. Cells were grown for 6, 12 and 24 h time points and cell pellets were collected for downstream applications (RNA extraction, qPCR, outlined in respective subsections). For confocal microscopy experiments, HTR8 cells were grown on glass coverslips (Thermo Fisher Scientific, Canada) to 20-30% confluence in 6-well tissue culture plates (Thermo Fisher Scientific, Canada). Cells were fixed in 100% methanol pre-chilled at −20°C for 5 mins and washed with ice-cold PBS. Cells were permeabilized for 10 mins using 0.1% Triton X-100 (Sigma-Aldrich, Canada). Blocking was performed using 1% BSA and 22.52 mg/mL glycine in PBS-T (supplemented with 0.1% Tween-20) for 1 h. Immunofluorescence protocol on cultured cells was performed as per Abcam using 1 μg/ml of anti-TTP rabbit polyclonal primary antibody incubated overnight (ab33058. Abcam Plc, Cambridge, UK). Donkey anti-rabbit Alexafluor 488 tagged secondary antibody was incubated for 1 hour on all slides (A-21206, Thermo Fisher Scientific, Canada) and DAPI was added during time of mounting. Confocal microscopy was performed using Leica SP2 Laser Scanning Confocal Microscope (Leica, Germany) using solid state laser line for 488nm excitation.

### Statistical analyses

Staining intensity (PARP) and differential cell counting experiments (NF-κB and TTP Immunofluorescence and Immunohistochemistry experiments) were compared using non-parametric student's *t*-test followed by Mann-Whitney post-hoc test using Graphpad PRISM 6.01 software (GraphPad Software Inc., California, USA) to compare between LPS and control placenta groups. All *in vivo* Quantitative real-time PCR and Western blot data was compared by un-paired student's *t*-tests. The *in vitro* data generated using quantitative real-time PCR was only analyzed per time group, and not between time points. Due to this, a one-way Analysis of Variance (ANOVA) was used to compare *in vitro* qPCR findings. A *P* value of < 0.05 between groups and results was considered statistically significant.

## SUPPLEMENTARY MATERIALS FIGURES


