# Supplementary material for: RNA binding protein, tristetraprolin in a murine model of recurrent pregnancy loss
Source: Oncotarget. 2016 Oct 9;7(45):72486–502. doi: 10.18632/oncotarget.12539 (PMC5341924; doi:10.18632/oncotarget.12539)
Supplement: Supplementary file 1 [file oncotarget-07-72486-s001.pdf]

RNA binding protein, tristetraprolin in a murine model of recurrent pregnancy loss

Supplementary Material

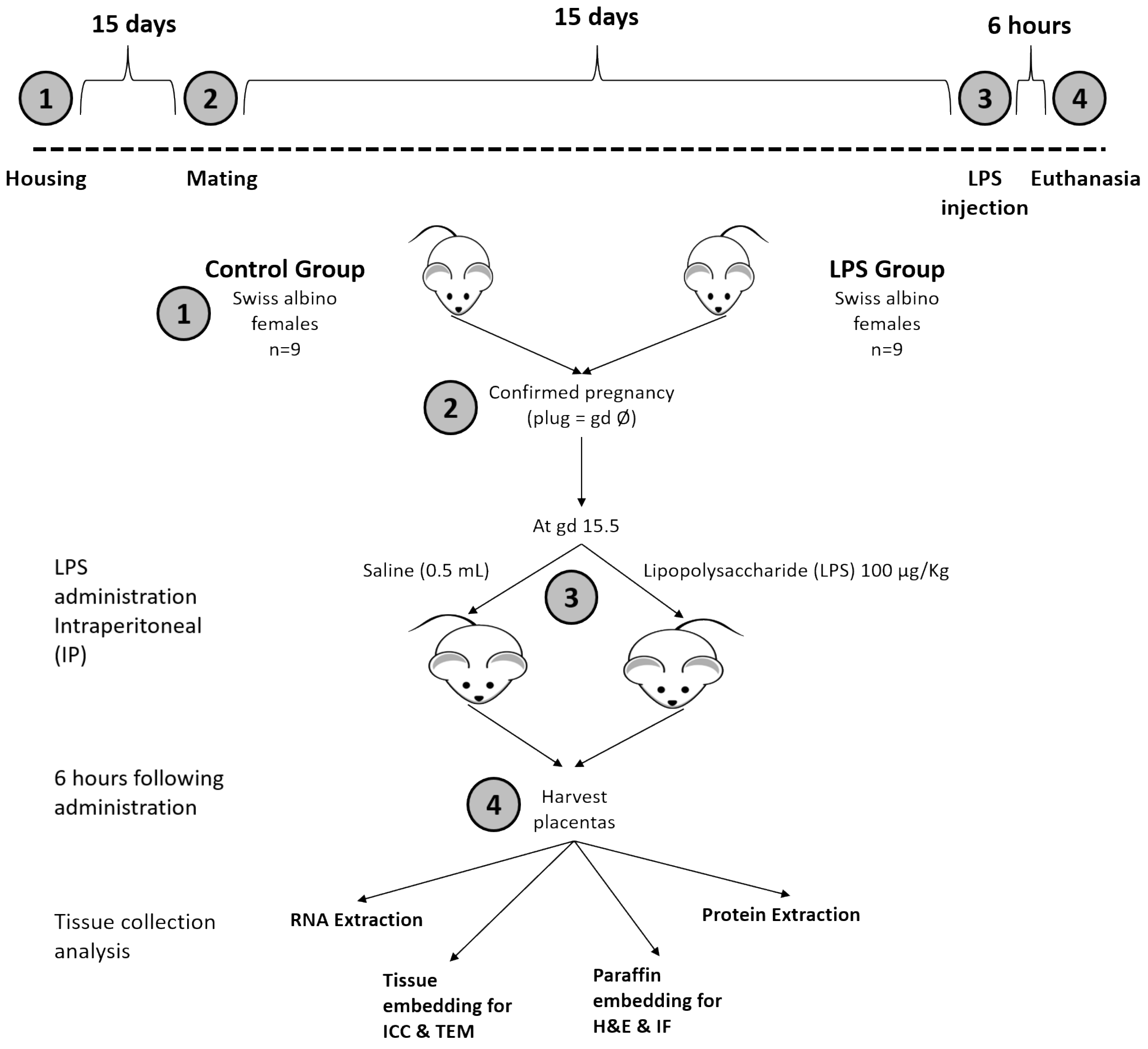

Supplemental Figure 1. Experimental outline of investigation into effects on RNA binding proteins in LPS treated placentas in a murine model of RPL. Swiss albino mice were separated into control (n=9) and LPS (n=9) groups (outlined as #1). Breeding pairs were set up for both groups of mice (outlined as #2). Upon 15.5 days of pregnancy (detection by vaginal plug), mice were either administered with 100 µg/Kg of LPS (LPS group), or 0.5 mL of Saline

(control group) via Intraperitoneal (IP) injection (outlined as #3). Six hours following administration, mice from both groups were sacrificed and placentas and tissues were harvested and utilized for downstream analyses (RNA and protein extraction as well as embedding for immunohistochemical techniques; outlined as #4).

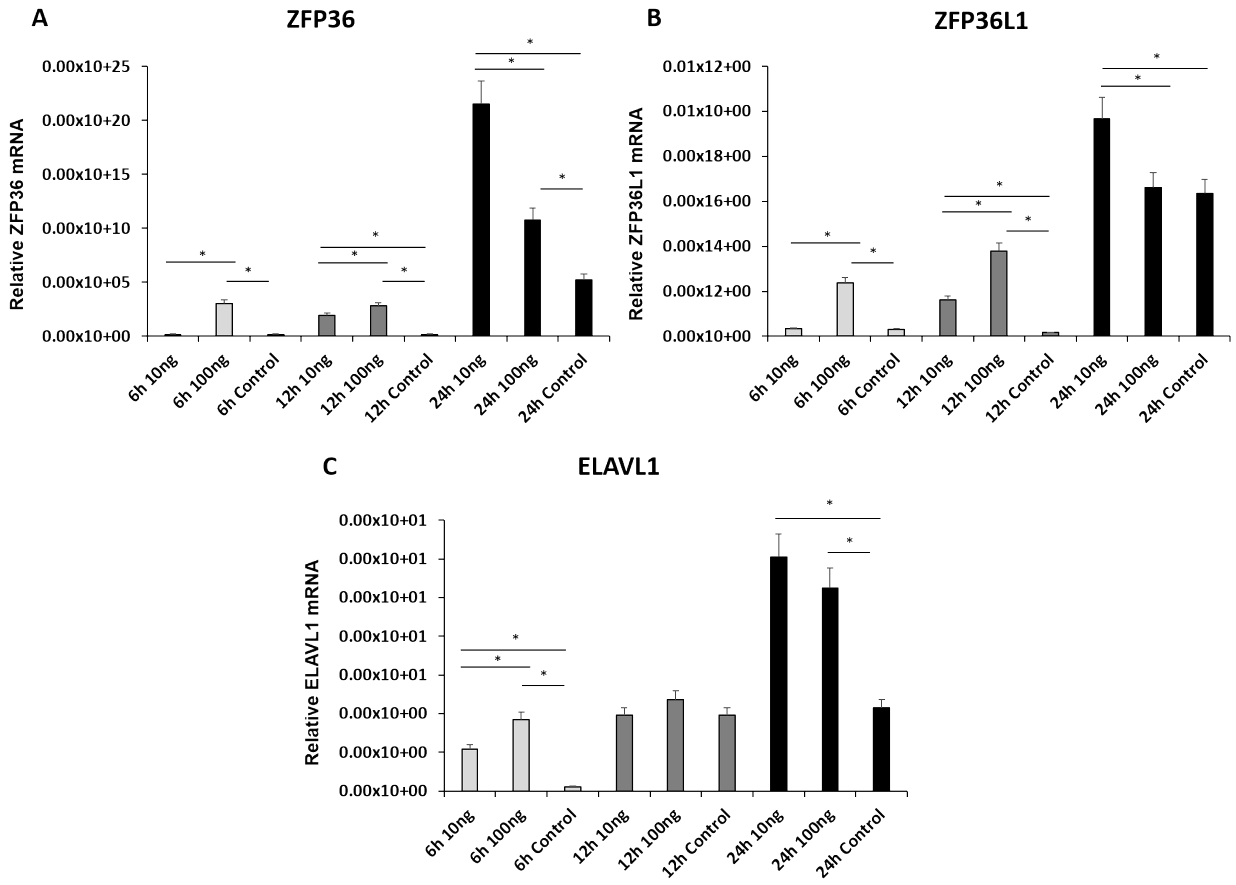

Supplemental Figure 2. ZFP36, ZFP36L1 and ELAV like RNA binding protein 1 (*ELAVL1*) mRNA expression is significantly upregulated in *in vitro* HTR8 SVneo trophoblast cells treated with LPS compared to control. Transcripts for *ELAVL1, ZFP36, and ZFP36L1* were measured in our *in vitro* model LPS stimulated HTR8 cells and compared with control at 6 h, 12 h and 24 h time points. Transcript levels of *ZFP36, ZFP36L1 and ELAVL1* were significantly upregulated at 6 h at 100 ng treatments (Panels A-C). Only *ELAVL1*

was significantly upregulated at 6 h 10 ng treatment group compared to its respective group (Panel C) Both *ZFP36* and *ZFP36L1* were also significantly upregulated at 12 h for both 10 ng and 100 ng treatments for (Panels A and B). Transcript levels for all three genes were significantly upregulated at 24 h at both doses, with the exception of *ZFP36L1*, which differed only at 10 ng (Panels A-C). All data was normalized and expressed relative to GAPDH housekeeping gene. Expression data illustrated as mean +/- SEM. * $P < 0.05$.

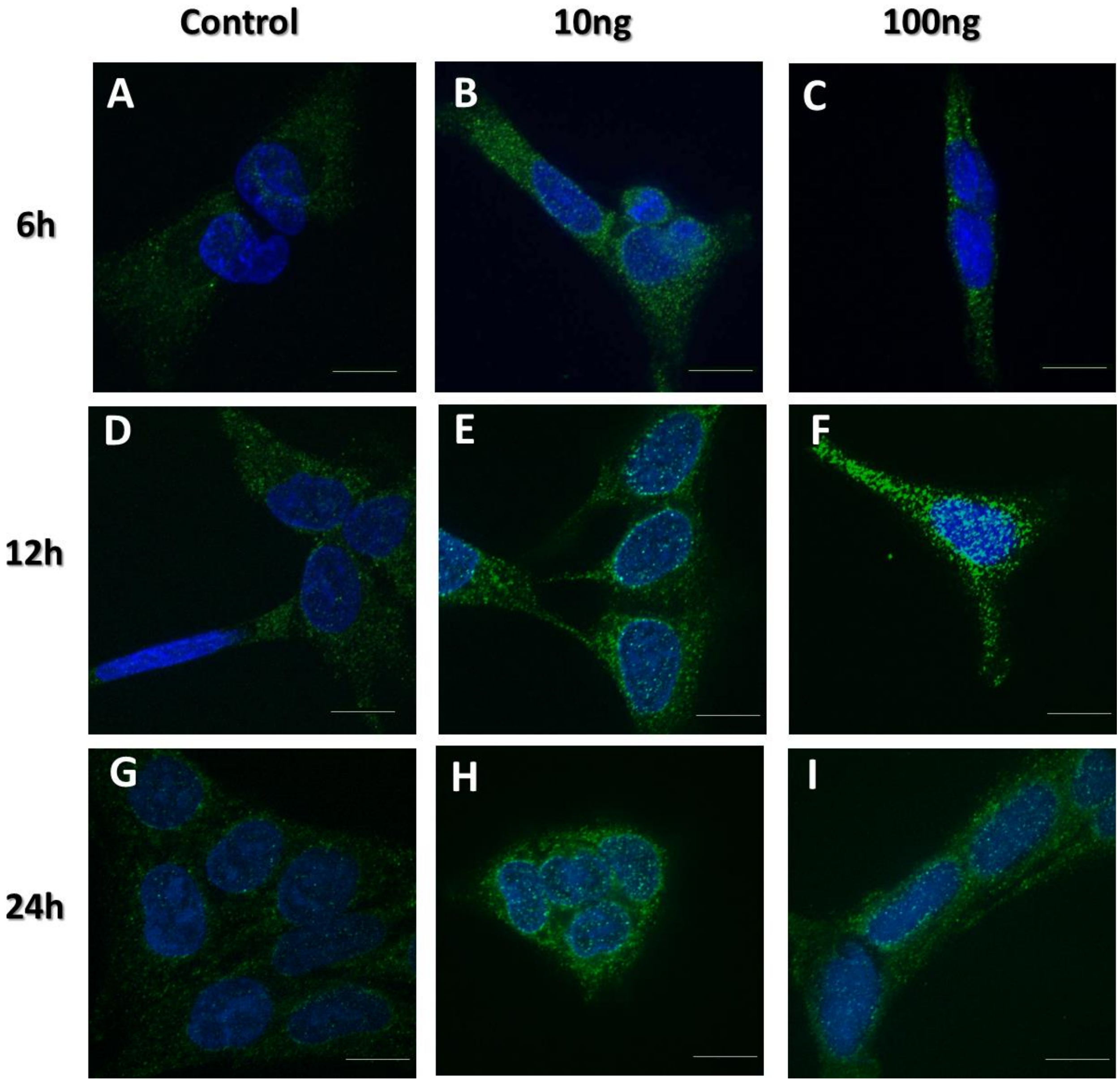

Supplemental Figure 3. Laser scanning confocal microscopy of TTP immunolocalization in the nucleus and cytoplasm of *in vitro* HTR8 SVneo trophoblast cells treated with LPS compared to control. Higher nuclear as well as cytoplasmic TTP were detected in 10 ng (Panels B,E,H) and 100 ng (Panels C,F,I) of LPS in HTR8 trophoblast cells compared to their respective time controls (Panels A,D,G). Higher TTP fluorescence was detected in 100 ng LPS treated group of HTR8 trophoblast cells when treated for longer 12 h and 24 h time points (Panels F compared to D and I compared to G). Original magnification 15 μm.
